# Risk Factors Associated with Structural Progression in Normal-Tension Glaucoma: Intraocular Pressure, Systemic Blood Pressure, and Myopia

**DOI:** 10.1167/iovs.61.8.35

**Published:** 2020-07-27

**Authors:** Kwanghyun Lee, Heon Yang, Joo Yeon Kim, Gong Je Seong, Chan Yun Kim, Hyoung Won Bae

**Affiliations:** 1 Institute of Vision Research, Department of Ophthalmology, Yonsei University College of Medicine, Seoul, South Korea; 2 Department of Ophthalmology, National Health Insurance Service Ilsan Hospital, Goyang, Gyeonggi-do, South Korea; 3 Kong Eye Clinic, Seoul, South Korea

**Keywords:** ganglion cell inner plexiform layer, normal-tension glaucoma, optical coherence tomography, glaucoma progression, retinal nerve fiber layer

## Abstract

**Purpose:**

To determine risk factors associated with structural progression in medically treated normal-tension glaucoma (NTG).

**Methods:**

This retrospective cohort study included 166 NTG patients (average age, 56.5 years; average mean deviation, −4.2 dB). The structural progression endpoint was determined by optical coherence tomography; significant thickness differences in the peripapillary retinal nerve fiber layer (RNFL) or macular ganglion cell inner plexiform layer (GCIPL) that exceeded baseline test-retest variability were identified with event-based guided-progression analysis. Intraocular pressure and systemic blood pressure (BP) were measured at each visit throughout the follow-up period, and the risk for progression was evaluated with Cox regression. Myopic disc features and antihypertensives were also analyzed. Tree analysis was used to determine the cutoff values and elucidate influential risk factors.

**Results:**

Structural progression, defined as progressive peripapillary RNFL or macular GCIPL thinning, was identified in 62 eyes. Occurrence of disc hemorrhages, presence of diabetes, and lower minimum systolic BP were associated with progression (hazard ratio [HR]: 2.116, *P* = 0.005; HR: 1.998, *P* = 0.031; HR: 0.968, *P* = 0.005; respectively). The cutoff value derived from the tree analysis of minimum systolic BP was 108 mm Hg. The tree analysis revealed systolic and diastolic BP to be the most influential risk factors for progressive peripapillary RFNL thinning and progressive macular GCIPL thinning, respectively.

**Conclusions:**

Low BP measured during follow-up correlated with structural progression in medically treated NTG eyes, indicating that the evaluation of hypotension is required during the management of NTG patients. The tree analysis identified BP target values that may help prevent glaucoma progression.

Normal-tension glaucoma (NTG) is a type of progressive glaucomatous optic neuropathy associated with a normal range of intraocular pressure (IOP).^1^ Although IOP is considered to be the most significant risk factor for NTG progression, several others, including aberrant systemic and ocular hemodynamics, have also been reported to influence the development and progression of NTG.^2^^–^^4^ The effect of myopia on NTG progression, however, remains controversial.^5^^,^^6^

Although standard automated perimetry (SAP) is most commonly used as a periodic visual field (VF) examination to monitor the progression of glaucoma, SAP is limited by its subjectivity and its sensitivity to short- or long-term fluctuations in results^7^; by contrast, the use of optical coherence tomography (OCT) to perform a structural progression analysis benefits from superior objectivity and ease of interpretation. Hence, temporal changes in the retinal nerve fiber layer (RNFL) or ganglion cell-inner plexiform layer (GCIPL) thickness measured with OCT feature widespread application as indices of the structural progression of glaucoma.^8^^,^^9^ In addition, structural progression detected by OCT can reportedly predict VF progression with better repeatability and reproducibility than can the VF test.^10^

This study aimed to evaluate the effect of IOP, myopic disc change, and systemic blood pressure (BP) on the structural progression of NTG. Decision tree analysis was used to determine the cutoff value and significance of each parameter. This study also examined the association between antihypertensive medication and structural progression. We expect these analyses to yield target values that will help to prevent glaucoma progression.

## Methods

This study was approved (4-2019-0835) by the Institutional Review Board of Severance Hospital, Yonsei University College of Medicine and adhered to the Declaration of Helsinki. We retrospectively reviewed the medical records of all the patients who visited the glaucoma clinic at our institution from November 2012 to July 2019. The requirement for informed consent was waived owing to our study's retrospective design and anonymization of clinical data.

All participants underwent complete ophthalmic examinations: the measurements of best-corrected visual acuity, IOP with a Goldmann applanation tonometer, and central corneal thickness; slit-lamp biomicroscopy; dilated fundus examination; color disc photography (Carl Zeiss Meditec, Jena, Germany); spectral-domain OCT (Cirrus HD-OCT, software v11.0, Carl Zeiss Meditec); axial-length (AL) measurement (IOL Master, Carl Zeiss Meditec); and a VF test (Humphrey Field Analyzer II; Carl Zeiss Meditec). Patients were followed up every six months for at least two years, and OCT examinations were performed once or twice a year. IOP and BP (right arm, sitting) were measured at each visit. Based on standard clinical protocol, all subjects were seated in a quiet location for at least five minutes before the measurements.

The mean IOP was calculated by averaging all the measurements during the follow-up period. If patients underwent cataract surgery, all postoperative IOP measurements obtained during the six weeks after the surgery were excluded to avoid the inclusion of transient IOP changes. The standard deviation (SD) of IOP measurements was used to define IOP fluctuation (SD IOP). Peak IOP was the single highest measurement recorded during the entire follow-up. Mean systolic BP (SBP) and diastolic BP (DBP) were calculated by averaging all the corresponding BP measurements. SBP and DBP fluctuations were defined using the SD of BP measurements. Maximum and minimum SBP or DBP were the highest and lowest corresponding single measurements during the entire follow-up, respectively. Mean arterial pressure (MAP) was calculated as 1/3 mean SBP + 2/3 mean DBP. Mean ocular perfusion pressure (MOPP) was calculated as 2/3 MAP − mean IOP. Systolic perfusion pressure (SPP) was calculated as the mean SBP − mean IOP, and diastolic perfusion pressure (DPP) was calculated as the mean DBP − mean IOP. When structural progression was detected, only IOP and BP measurements obtained before the detection were included in subsequent analyses.

Data on antihypertensive-medication use were obtained when available from electronic health records. Antihypertensive medications were categorized into four classes: diuretics, β-blockers, calcium channel blockers (CCBs), and angiotensin II receptor blockers.

The inclusion criteria included a best-corrected visual acuity of 20/40 or better and an open angle on gonioscopy. NTG was diagnosed when the maximum untreated IOP was < 21 mm Hg on three repeated measurements obtained at different times on separate follow-up visits and in the presence of glaucomatous optic discs (neuroretinal rim thinning and excavation) and peripapillary RNFL defect—regardless of the presence or absence of glaucomatous VF defects. Individuals with the following were excluded: (1) secondary causes of glaucomatous optic neuropathy, (2) history of glaucoma (including glaucoma filtration surgery) or refractive surgery, and (3) neurologic or systemic diseases influencing OCT measurements. Because our preliminary data showed a potential floor effect for RNFL and GCIPL (Supplementary Fig. S1), we also excluded eyes with a mean deviation (MD) < −20 dB and RNFL and GCIPL thicknesses < 65 µm. If both eyes were eligible for inclusion, one of the participant's eyes was randomly selected.

### Measurement of Parameters Related to Optic-Disc Tilt and Torsion

Color fundus images were evaluated by two investigators (H.Y. and K.L.) in a masked fashion using ImageJ software (v1.52; National Institutes of Health, Bethesda, MD, USA). The disc-tilt ratio was defined as the ratio between the longest and shortest diameters of the optic disc. Optic-disc torsion was defined as the deviation angle of the long axis of the optic disc from the vertical meridian: the vertical line situated 90° from the line that connects the fovea and the center of the optic disc.^11^ The average measurements of the two investigators were used in the final analysis.

### Optical Coherence Tomography

The OCT images of the peripapillary RNFL and macular GCIPL were obtained with optic disc cube and macular scans, respectively, using a Cirrus HD-OCT. The optic disc cube scan produced an RNFL thickness map of 6 × 6 mm (200 × 200 pixels) in area centered on the optic nerve head. The peripapillary RNFL thickness was measured circularly with a diameter of 3.46 mm. The macular cube scan generated a GCIPL thickness map of 6 × 6 mm (512 × 128 pixels) in area centered on the fovea. The macular GCIPL thickness was measured in the annulus with inner vertical and horizontal diameters of 1 and 1.2 mm, respectively, and outer vertical and horizontal diameters of 4 and 4.8 mm, respectively. At least five reliable OCT scans from separate visits were required for study inclusion. All OCT scans had a signal strength of ≥6. Scans with motion artefacts, poor centration, or missing data were excluded.

### Guided Progression Analysis of the Ganglion Cell-Inner Plexiform Layer and Retinal Nerve Fiber Layer

We evaluated the structural progression of peripapillary RNFL and macular GCIPL using an event-based algorithm provided by guided progression analysis (GPA). The GPA algorithm compared the changes in peripapillary RNFL and macular GCIPL thicknesses at individual superpixels (1 superpixel = 4 × 4 pixels) between the follow-up and two baseline thickness maps. For the change to be classified as significant, a change of at least 20 adjacent superpixels must be detected in the RNFL or GCIPL thickness maps. If a follow-up OCT examination demonstrated a statistically significant difference in thickness that exceeded the baseline test-retest variability, the superpixel was labeled yellow to indicate possible loss; if confirmed on a second follow-up OCT examination, it was labeled red to signal probable loss. Progressive thinning of peripapillary RNFL and macular GCIPL was defined as a “likely loss” in the event analysis during follow-up, with the same changes being observed in the most recent follow-up visit.

### Perimetry

SAP was performed using the Swedish interactive threshold algorithm standard 24-2 program in the Humphrey Field Analyzer II. Only reliable VF test results (false-positive errors < 15%, false-negative errors < 15%, and fixation loss < 20%) were included.

### Decision Tree Analysis

The decision tree analysis model is a statistical tool used to separate a group into two subgroups based on individual risk factors. The decision tree analysis was performed using the ctree function in the party package of R, which enables conditional inference tree analysis.^12^ Briefly, conditional inference tree analysis generates a decision tree by recursively partitioning the population of interest into subgroups. At each partition, it searches for the best predictor and corresponding cutoff value that splits one group into two subgroups such that the response is significantly different between the two groups. Because multiple predictors are considered, Bonferroni correction is used to counteract multiple comparisons.^13^ Traditional decision tree analysis, such as classification and regression trees, selects variables that maximize information measurement (Gini coefficient or information gain). This method can produce complex trees, called overfits, that are not generated well from training data. Therefore some techniques such as pruning are needed to avoid this problem. However, the conditional inference tree analysis used in this study benefits from not requiring such a technique, because this method uses statistical theory (selection by permutation-based significance testing) and thereby removes the potential bias in classification and regression trees or similar decision trees.^13^ Factors that were found to be associated with structural progression in the univariable Cox analysis were used as the input variables.

### Statistical Analysis

Statistical analysis was performed using the R software v3.6.0 (R Foundation for Statistical Computing, Vienna, Austria). Baseline clinical variables are presented as mean ± SD. Structural progression was categorized into at least one of three groups: progressive peripapillary RNFL or macular GCIPL thinning, progressive peripapillary RNFL thinning, progressive macular GCIPL thinning. Univariable and multivariable Cox regression was used to calculate the hazard ratios (HRs) of clinical variables for each structural progression. HR was described as a mean with a 95% confidence interval. To avoid multicollinearity complications, we calculated the variance inflation factor and excluded variables with values of above 2.5. Because DBP and SBP correlated with each other, we excluded one variable with the Akaike information criterion calculation when both variables were included in the multivariate analysis. Student's *t*-test was used to compare the clinical variables between two groups according to history of hypertension (HTN) or antihypertensive medication use. Decision tree analysis was performed as described above. Kaplan-Meier analysis was used to compare the progression rate between groups defined by the decision tree analysis. We also used Cox regression to calculate the HRs of the groups defined by the tree analysis. All tests reported *P* values as bilateral; those of less than 0.05 were considered statistically significant.

## Results

We identified 213 eyes of 213 NTG patients and selected 166 eyes based on inclusion and exclusion criteria. The baseline means of age, AL, and IOP were 56.3 ± 15.3 years, 24.3 ± 1.7 mm, and 14.9 ± 2.4 mm Hg, respectively. Clinical variables including baseline peripapillary RNFL thickness, macular GCIPL thickness, and MD are described in Table 1. The mean follow-up period was 48.9 ± 15.2 months. Structural progression was identified in 62 eyes: 21, only progressive peripapillary RNFL thinning; 18, only progressive macular GCIPL thinning; 23, both peripapillary RNFL and macular GCIPL thinning.

**Table 1.** Participant Demographics and the Comparisons of Variables Between Stable and Progressed Eyes

	Total (n = 166)	Stable (n = 104)	Progressed (n = 62)	*P* *
Age, years	56.3 ± 15.3	57.3 ± 14.2	54.8 ± 16.9	0.309
Hypertension, n (%)	54 (32.5%)	30 (28.8%)	24 (38.7%)	0.254
Diabetes mellitus, n (%)	24 (14.5%)	11 (10.6%)	13 (21.0%)	0.107
Central corneal thickness (µm)	536.0 ± 37.0	538.2 ± 32.5	532.2 ± 44.0	0.394
Disc hemorrhage, n (%)	44 (26.5%)	19 (18.3%)	25 (40.3%)	**0.003**
RNFL thickness, µm	80.8 ± 8.9	79.9 ± 8.6	82.4 ± 9.3	0.085
GCIPL thickness, µm	74.5 ± 5.7	74.5 ± 5.9	74.6 ± 5.5	0.914
Mean deviation, dB	−4.2 ± 3.8	−4.1 ± 3.3	−4.5 ± 4.6	0.507
Axial length, mm	24.3 ± 1.7	24.1 ± 1.5	24.6 ± 1.8	0.252
Disc torsion, degrees	0.5 ± 8.0	0.7 ± 7.4	0.3 ± 8.9	0.806
Disc tilt ratio	1.2 ± 0.2	1.1 ± 0.2	1.2 ± 0.2	0.078
Baseline IOP, mm Hg	14.9 ± 2.4	14.8 ± 2.3	15.0 ± 2.7	0.624
Mean IOP, mm Hg	13.0 ± 1.8	13.0 ± 1.9	13.0 ± 1.7	0.988
SD IOP, mm Hg	1.6 ± 0.5	1.5 ± 0.4	1.7 ± 0.5	**0.034**
Peak IOP, mm Hg	15.6 ± 2.2	15.4 ± 2.2	15.9 ± 2.1	0.117
Mean SBP, mm Hg	121.8 ± 12.4	123.2 ± 12.8	119.4 ± 11.5	0.054
Minimum SBP, mm Hg	109.0 ± 13.4	110.5 ± 13.8	106.4 ± 12.4	0.055
Maximum SBP, mm Hg	134.8 ± 14.2	135.9 ± 14.3	133.1 ± 13.9	0.229
SD SBP, mm Hg	10.1 ± 4.6	10.2 ± 4.9	10.0 ± 4.1	0.727
Mean DBP, mm Hg	71.1 ± 8.3	71.8 ± 8.6	69.9 ± 7.8	0.148
Maximum DBP, mm Hg	80.7 ± 9.7	81.4 ± 9.5	79.7 ± 10.0	0.274
Minimum DBP, mm Hg	61.8 ± 9.4	62.8 ± 9.9	60.1 ± 8.5	0.084
SD DBP, mm Hg	7.4 ± 3.0	7.4 ± 3.0	7.4 ± 3.0	0.973
MAP, mm Hg	88.0 ± 9.3	88.9 ± 9.5	86.4 ± 8.6	0.085
MOPP, mm Hg	50.0 ± 6.1	50.6 ± 6.2	48.9 ± 6.0	0.082
SPP, mm Hg	108.7 ± 12.4	110.2 ± 12.5	106.3 ± 11.9	0.054
DPP, mm Hg	58.0 ± 8.2	58.8 ± 8.3	56.8 ± 8.0	0.143

Parameters are presented as the mean ± standard deviation or n (%).

* *P* values were calculated with *t*-tests. Indicated in bold type, *P* < 0.05 indicates statistical significance.

### Identification of Factors Associated with Structural Progression

The univariable Cox regression analysis identified diabetes, disc hemorrhage, SD IOP, mean SBP, minimum SBP, mean DBP, minimum DBP, MAP, MOPP, SPP, and DPP as being associated with structural progression when defined as progressive peripapillary RNFL or macular GCIPL thinning (Table 2). The multivariable Cox regression analysis revealed diabetes, disc hemorrhage, and minimum SBP to be associated with structural progression (Table 2). SD IOP was associated with structural progression after having adjusted for diabetes and disc hemorrhage (HR = 1.784 [1.031–3.084], *P* = 0.038).

**Table 2.** Univariable and Multivariable Cox Analysis of Progressive Peripapillary RNFL or Macular GCIPL Thinning

	Univariable Cox Analysis			Multivariable Cox Analysis		
	Hazard Ratio	95% CI	*P*	Hazard Ratio	95% CI	*P*
Age, years	1.001	0.984–1.018	0.921			
Hypertension	1.612	0.960–2.707	0.071			
Diabetes mellitus	1.971	1.062–3.656	**0.031**	1.998	1.067–3.742	**0.031**
Central corneal thickness (µm)	0.999	0.9991–1.006	0.736			
Disc hemorrhage	2.191	1.309–3.667	**0.003**	2.116	1.260–3.555	**0.005**
RNFL thickness, µm	1.013	0.986–1.040	0.353			
GCIPL thickness, µm	0.987	0.943–1.034	0.584			
Mean deviation, dB	0.977	0.915–1.044	0.500			
Axial length, mm	0.967	0.780–1.198	0.758			
Disc torsion, degrees	0.982	0.950–1.015	0.279			
Disc tilt ratio	1.250	0.485–3.222	0.644			
Baseline IOP, mm Hg	0.968	0.865–1.083	0.566			
Mean IOP, mm Hg	0.982	0.843–1.145	0.820			
SD IOP, mm Hg	1.798	1.034–3.127	**0.038**	1.417	0.794–2.532	0.239
Peak IOP, mm Hg	1.028	0.911–1.160	0.656			
Mean SBP, mm Hg	0.965	0.943–0.987	**0.002**			
Minimum SBP, mm Hg	0.963	0.942–0.984	**0.001**	0.968	0.947–0.990	**0.005**
Maximum SBP, mm Hg	0.984	0.965–1.004	0.119			
SD SBP, mm Hg	1.011	0.955–1.071	0.709			
Mean DBP, mm Hg	0.956	0.924–0.990	**0.012**			
Maximum DBP, mm Hg	0.979	0.951–1.007	0.132			
Minimum DBP, mm Hg	0.967	0.943–0.991	**0.007**			
SD DBP, mm Hg	1.009	0.931–1.094	0.826			
MAP, mm Hg	0.954	0.925–0.985	**0.004**			
MOPP, mm Hg	0.934	0.891–0.980	**0.005**			
SPP, mm Hg	0.966	0.945–0.988	**0.003**			
DPP, mm Hg	0.957	0.924–0.991	**0.014**			

Indicated in bold type, *P* < 0.05 indicates statistical significance. CI, confidence interval.

Disc hemorrhage, SD IOP, mean SBP, minimum SBP, minimum DBP, and SPP were associated with structural progression when defined as progressive peripapillary RNFL thinning in univariable Cox regression analysis; disc hemorrhage and minimum SBP remained associated with progressive peripapillary RNFL thinning in the multivariable Cox regression analysis (Table 3).

**Table 3.** Univariable and Multivariable Cox Analysis of Progressive Peripapillary RNFL Thinning

	Univariable Cox Analysis			Multivariable Cox Analysis		
	Hazard Ratio	95% CI	*P*	Hazard Ratio	95% CI	*P*
Age, years	0.984	0.965–1.003	0.100			
Hypertension	1.050	0.560– 1.968	0.879			
Diabetes mellitus	1.410	0.669–2.972	0.367			
Central corneal thickness (µm)	1.004	0.995–1.014	0.337			
Disc hemorrhage	2.535	1.387–4.631	**0.002**	2.523	1.367–4.655	**0.003**
RNFL thickness, µm	1.032	0.999–1.065	0.054			
GCIPL thickness, µm	0.969	0.914–1.026	0.282			
Mean deviation, dB	1.058	0.971–1.152	0.198			
Axial length, mm	1.114	0.887–1.399	0.355			
Disc torsion, degrees	0.990	0.952–1.030	0.634			
Disc tilt ratio	1.451	0.540–3.897	0.460			
Baseline IOP, mm Hg	1.057	0.935–1.196	0.376			
Mean IOP, mm Hg	0.994	0.802–1.136	0.599			
SD IOP, mm Hg	2.253	1.159–4.379	**0.017**	1.473	0.733–2.962	0.277
Peak IOP, mm Hg	1.013	0.880–1.166	0.857			
Mean SBP, mm Hg	0.967	0.941–0.994	**0.016**			
Minimum SBP, mm Hg	0.954	0.929–0.979	**<0.001**	0.956	0.930–0.983	**0.002**
Maximum SBP, mm Hg	0.990	0.967–1.013	0.383			
SD SBP, mm Hg	1.044	0.979–1.112	0.189			
Mean DBP, mm Hg	0.973	0.935–1.013	0.177			
Maximum DBP, mm Hg	0.963	0.934–0.993	**0.017**			
Minimum DBP, mm Hg	0.997	0.968–1.028	0.865			
SD DBP, mm Hg	1.073	0.976–1.180	0.146			
MAP, mm Hg	0.965	0.930–1.001	0.057			
MOPP, mm Hg	0.953	0.902–1.006	0.080			
SPP, mm Hg	0.970	0.944–0.996	**0.022**			
DPP, mm Hg	0.976	0.938–1.015	0.229			

Indicated in bold type, *P* < 0.05 indicates statistical significance. CI, confidence interval.

Disc hemorrhage, mean SBP, minimum SBP, mean DBP, minimum DBP, maximum DBP, MAP, MOPP, SPP, and DPP were associated with structural progression when defined as progressive macular GCIPL thinning in the univariable Cox regression analysis; disc hemorrhage and minimum DBP remained associated with progressive macular GCIPL thinning in the multivariable Cox regression analysis (Table 4).

**Table 4.** Univariable and Multivariable Cox Analysis of Progressive Macular GCIPL Thinning

	Univariable Cox Analysis			Multivariable Cox Analysis		
	Hazard Ratio	95% CI	*P*	Hazard Ratio	95% CI	*P*
Age, years	1.007	0.986–1.028	0.522			
Hypertension	1.664	0.882–3.140	0.116			
Diabetes mellitus	1.500	0.689–3.264	0.307			
Central corneal thickness (µm)	0.996	0.988–1.004	0.356			
Disc hemorrhage	2.244	1.183–4.256	**0.013**	1.983	1.037–3.790	**0.038**
RNFL thickness, µm	1.009	0.975–1.044	0.611			
GCIPL thickness, µm	0.997	0.944–1.053	0.924			
Mean deviation, dB	0.934	0.870–1.003	0.062			
Axial length, mm	0.938	0.717–1.226	0.639			
Disc torsion, degrees	0.994	0.955–1.034	0.752			
Disc tilt ratio	1.611	0.546–4.753	0.387			
Baseline IOP, mm Hg	0.963	0.838–1.106	0.594			
Mean IOP, mm Hg	1.008	0.837–1.214	0.935			
SD IOP, mm Hg	1.689	0.860–3.319	0.128			
Peak IOP, mm Hg	1.020	0.876–1.186	0.801			
Mean SBP, mm Hg	0.965	0.939–0.993	**0.014**			
Minimum SBP, mm Hg	0.969	0.944–0.994	**0.016**			
Maximum SBP, mm Hg	0.982	0.959–1.006	0.134			
SD SBP, mm Hg	0.986	0.917–1.061	0.708			
Mean DBP, mm Hg	0.940	0.899–0.982	**0.005**			
Maximum DBP, mm Hg	0.961	0.933–0.990	**0.008**			
Minimum DBP, mm Hg	0.964	0.930–0.999	**0.042**	0.965	0.936–0.995	**0.022**
SD DBP, mm Hg	0.984	0.888–1.089	0.750			
MAP, mm Hg	0.946	0.909–0.984	**0.005**			
MOPP, mm Hg	0.920	0.868–0.976	**0.006**			
SPP, mm Hg	0.966	0.940–0.993	**0.015**			
DPP, mm Hg	0.938	0.897–0.981	**0.005**			

Indicated in bold type, *P* < 0.05 indicates statistical significance. CI, confidence interval.

### Effect of Antihypertensives on Structural Progression

Subgroup analysis of patients with HTN was performed to evaluate the effect of antihypertensives on structural progression. Compared to participants without HTN, those with HTN were older (*P* < 0.001) and had a more extensive history of diabetes (*P* < 0.001) and shorter axial length (*P* = 0.002, Supplementary Table S1). Data on antihypertensive medication prescriptions were available for 34 patients. Twenty-two patients used angiotensin II receptor blockers; 18, CCB; 11, β-blockers; and six, diuretics. Eighteen patients used only one type of drug, whereas 16 patients used two or more. The univariable Cox regression analysis revealed a significant association between CCB prescription and structural progression when defined as progressive peripapillary RNFL or macular GCIPL thinning (HR = 0.201 [0.052–0.773], *P* = 0.020, Supplemental Table S2). However, this effect did not remain significant after adjusting for mean SBP (*P* = 0.118, Supplemental Table S2). The results for peripapillary RNFL and macular GCIPL thinning are presented in Supplemental Tables S3 and S4.

### Decision Tree Analysis

**Figure 1.**
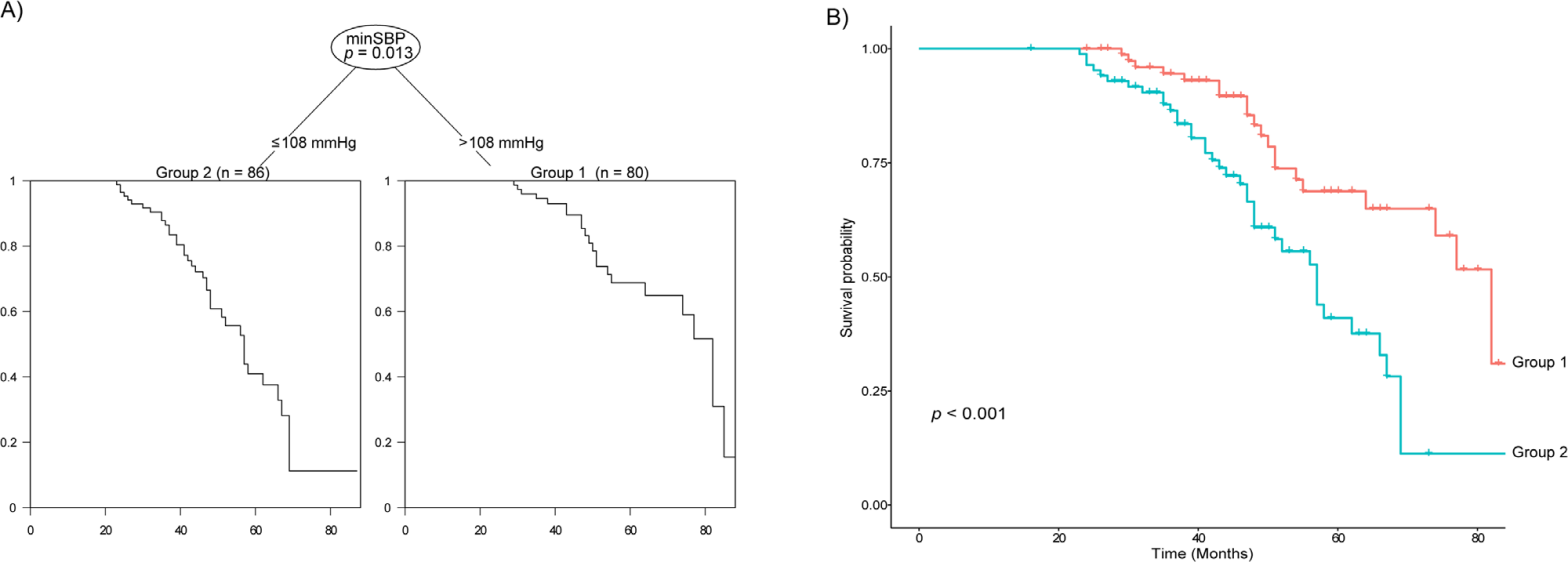
(**A**) Decision tree analysis for structural progression when defined as progressive peripapillary retinal nerve fiber layer or macular ganglion cell-inner plexiform layer thinning; (**B**) Kaplan-Meier analysis showing significant differences in the progression rate between the two groups defined by the tree analysis. MinSBP, minimum systolic blood pressure.

The clinical variables that were found to be significantly associated with structural progression in the univariable Cox regression analysis were used as possible risk factors in the decision tree analysis. When structural progression was defined as progressive peripapillary RNFL of macular GCIPL thinning, minimum SBP was the most important factor, with a cutoff value of 108 mm Hg (Fig. 1A). Kaplan-Meier analysis also showed statistically different rates of structural progression (Fig. 1B). Eyes with a minimum SBP ≤ 108 mm Hg showed more progression than did eyes with a minimum SBP < 108 mm Hg (HR 2.563 [1.494–4.396], *P* < 0.001).

**Figure 2.**
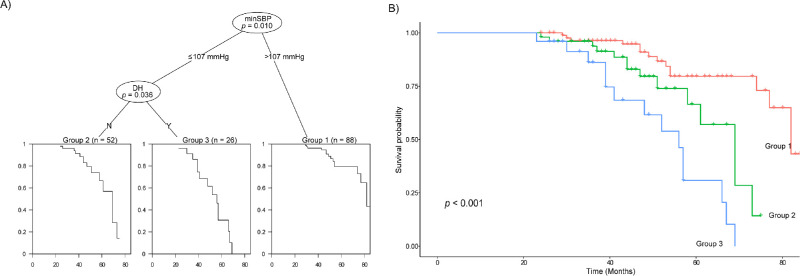
(**A**) Decision tree analysis for progressive peripapillary retinal nerve fiber layer thinning; (**B**) Kaplan-Meier analysis showing significant differences in the progression rate between the three groups defined by the tree analysis. MinSBP, minimum systolic blood pressure; DH, disc hemorrhage.

In terms of progressive peripapillary RNFL thinning, minimum SBP and disc hemorrhage were found to be the most significant factors (Fig. 2A). A minimum SBP > 107 mm Hg was associated with the least progression of peripapillary RNFL thinning. For the minimum SBP of ≤ 107 mmHg, disc hemorrhage was significantly associated with progressive peripapillary RNFL thinning. Kaplan-Meier analysis also showed statistically different rates of structural progression (Fig. 2B). Compared to a minimum SBP > 107 mm Hg, the HR of eyes without disc hemorrhage and a minimum SBP ≤ 107 mm Hg was 3.165 (1.435–6.981; *P* = 0.004), and that of eyes with disc hemorrhage and a minimum SBP ≤ 107 mm Hg was 7.602 (3.391–17.044; *P* < 0.001).

**Figure 3.**
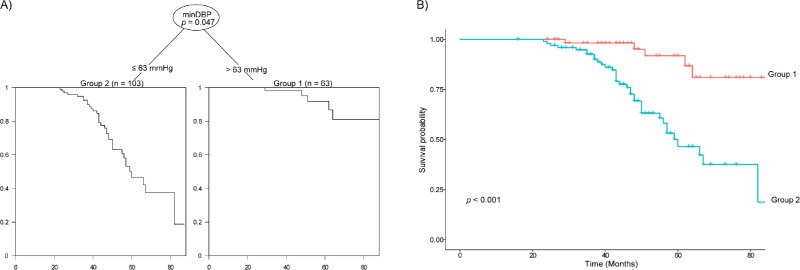
(**A**) Decision tree analysis for progressive macular GCIPL thinning; (**B**) Kaplan-Meier analysis showing significant differences in the progression rate between the two groups defined by the tree analysis. MinDBP, minimum diastolic blood pressure.

Minimum DBP was the most significant factor associated with progressive macular GCIPL thinning (Fig. 3A). Kaplan-Meier analysis revealed a difference in the rate of progressive macular GCIPL thinning between eyes with a minimum DBP > 63 mm Hg and those with ≤63 mm Hg (Fig. 3B). In contrast with eyes with a minimum DBP > 63 mm Hg (*P* < 0.001), the HR of eyes with a minimum DBP ≤ 63 mm Hg was 5.889 (2.297–15.096).

## Discussion

This study used tree analysis to identify and elucidate the significance of risk factors associated with the structural progression of NTG. Diabetes, disc hemorrhage, and minimum SBP were found to significantly influence structural progression. The tree analysis identified 108 mm Hg as the cutoff value for minimum SBP and revealed minimum SBP and DBP to be the most significant variables for progressive peripapillary RNFL thinning and progressive macular GCIPL thinning, respectively. Although CCB demonstrated a preventive effect in the eyes of patients with HTN, this effect was found to be nonsignificant after adjusting for BP parameters.

Previous studies have found an association between the incidence of glaucoma and low BP.^14^^,^^15^ Consistent with the present findings, Kaiser et al.^2^ showed that functional progression can occur in patients with low BP in patients with POAG and NTG, despite their IOP being well controlled. We have proposed cutoff (target) values for minimum SBP and DBP: 107–108 mm Hg and 63 mm Hg, respectively. Leske et al. ^14^ observed low SPP, DPP, and SBP to be associated with the incidence of glaucoma (relative risk ratios (RR): 0.91, 0.87, and 0.79 per 10 mm Hg, respectively). Individuals with an SPP < 98 mm Hg had a higher risk of glaucoma progression than those with an SPP > 153 mm Hg (RR, 2.0), and a DPP < 53 mm Hg was associated with a higher risk of glaucoma progression than was a DPP > 73 mm Hg (RR 2.1).^14^ Tham et al.^15^ found that, relative to individuals with an SBP of between 138 and 153 mm Hg, the odds ratio of those with an SBP < 124 mm Hg was 1.69 for primary open-angle glaucoma (POAG). Therefore the present investigation differs from previous studies in two ways: (1) our cutoff values for BP control were elucidated with decision tree analysis, and (2) we analyzed risk factors for the progression of glaucoma—not its incidence. Our findings indicate that hypotension is closely related to not only the development of glaucoma but also to its structural progression.

SBP and DBP seem to have greater effects on other structures of the eye. According to the tree analysis, minimum SBP featured a stronger association with progressive peripapillary RNFL thinning than did minimum DBP, which had a greater impact on progressive macular GCIPL thinning than did minimum SBP. Considering previous studies that revealed DBP to be primarily linked to tissue perfusion,^16^^–^^19^ our results suggest that macular GCIPL may be more critical to perfusion deficiency than to peripapillary RNFL. However, because we found a close correlation between SBP and DBP measurements, these results could be statistically different representations of the fact that systemic hypotension is associated with structural progression. Further research is needed to determine whether SBP and DBP differentially affect peripapillary RNFL and macular GCIPL.

IOP fluctuation is associated with structural progression, particularly progressive peripapillary RNFL thinning, but not with progressive macular GCIPL thinning. Mean IOP and peak IOP were not associated with any kind of structural progression. Although the relationship between long-term IOP fluctuation and glaucoma progression remains controversial,^20^^–^^24^ our results are consistent with those of the Advanced Glaucoma Intervention Study (AGIS): long-term IOP fluctuation was the most critical risk factor for VF progression in glaucoma.^22^ A univariable analysis performed by a large retrospective study of patients treated for POAG or primary angle closure glaucoma demonstrated that peak IOP and IOP fluctuation were significantly associated with disease progression; a multivariable analysis conducted by the same study revealed that only IOP fluctuation was significantly associated with disease progression.^21^ A retrospective chart review found a significant association between IOP fluctuation and glaucoma progression.^23^ However, these results contradict those of the Early Manifest Glaucoma Trial (EMGT), which observed that the mean IOP—not IOP fluctuation—was related to disease progression.^20^ These divergent results may be ascribed to differences in the research population; e.g., participants in the EMGT included untreated patients with early glaucoma.^24^ By contrast, the present investigation considered patients who were treated for diagnosed NTG. The mean IOP in our study was also lower than that reported by the EMGT (13.1 ± 1.7 vs. 20.7 ± 4.1 mm Hg). Above all, our study reflects the clinical situation in which the patient's treatment changes with their IOP—i.e., the subjects in this study were likely to have received additional medication when the mean IOP was considered elevated or a high peak IOP was observed. Our findings do not suggest that the average IOP is unrelated to structural progression but rather that IOP fluctuations could also affect glaucoma progression in clinical settings where treatment is altered to reach a target IOP. Hence, considering that the mean IOP of our participants was 13.0 mm Hg, our results recommend the reduction of IOP fluctuations even in patients with a mean IOP of ≤ 15 mm Hg.

Our study confirmed disc hemorrhage as a risk factor for NTG progression.^6^^,^^25^ In agreement with the results of previous studies,^6^^,^^26^^,^^27^ we found the HR of disc hemorrhage for structural progression defined as progressive peripapillary RNFL or macular GCIPL thinning to be 2.085 (progressive peripapillary RNFL thinning, 2.640; progressive macular GCIPL thinning, 2.205). While the etiology of disc hemorrhage is unclear, both mechanical^28^^,^^29^ and vascular mechanisms^30^^,^^31^ have been hypothesized. Quigley et al.^28^ suggested that disc hemorrhage occurs due to microvascular damage during posterior bowing of the lamina cribrosa. Sharpe et al.^32^ reported that laminar disinsertions were more frequently detected in glaucoma patients with disc hemorrhage. These results suggest that disc hemorrhage could be closely related to laminar changes in glaucoma patients. Hence, laminar damage can occur even if the IOP is well-controlled in patients with normal-tension glaucoma that might have caused the structural progression. On the other hand, some studies have reported that systemic vascular diseases, such as hypertension, diabetes mellitus, and atherosclerosis, can induce ischemic changes around the optic disc, increasing the incidence of disc hemorrhage.^33^^–^^35^ Furthermore, primary vascular dysregulation (i.e., Flammer syndrome) might be associated with disc hemorrhage in glaucoma patients.^30^ Similar to systemic hypotension, ischemic changes induced by a disc hemorrhage might therefore affect the structural progression of NTG eyes.

Previous studies have reported that diabetes contributes to an increased risk of developing open-angle glaucoma.^36^^–^^39^ These results are supported by evidence implicating impaired autoregulation in the development of glaucoma—especially normal-tension glaucoma.^40^^,^^41^ However, few studies have been published on the relationship between glaucoma progression and diabetes. In contrast with our own findings, the Advanced Glaucoma Intervention Study (AGIS) found no significant difference between progressive VF loss and diabetes.^22^ We excluded subjects with MD values of below −20.0dB, which may have resulted in a limited enrollment of participants with advanced glaucoma. Furthermore, whereas the AGIS evaluated VF changes, we investigated structural progression using OCT. Our results suggest that diabetes-induced glaucoma damage may be more evident in early glaucoma or may alter structure significantly more than function. Further study is required to confirm whether diabetes-induced changes in glaucoma differ according to the stage of glaucoma and whether such changes differentially affect structure and function.

Although myopia and myopic optic disc changes are reportedly associated with glaucoma progression,^42^^–^^44^ our study did not confirm these results. This controversy may be ascribed to differences between the study populations. Most studies that found an association between myopic optic disc changes and glaucoma progression included glaucomatous eyes with myopia. In contrast, the present study investigated NTG eyes regardless of myopia. The mean AL of our participants was 24.35 mm, which is relatively close to emmetropia. Our results suggest that NTG progression with myopia and myopic optic disc changes may significantly affect glaucomatous eyes with myopia but not glaucomatous eyes with emmetropia or hyperopia.

Univariable Cox regression analysis showed that CCB has a protective effect against glaucoma progression; because this association became nonsignificant in the multivariable Cox regression analysis, our results suggest that the protective effect of CCB may be related to BP. Prior research demonstrated the effect of CCB on VF improvements in NTG.^45^^,^^46^ In particular, one study reported that low doses of CCB could be used to treat vascular dysregulation in glaucomatous eyes,^47^ and another reported that low doses of CCB could be safer and more efficient than conventional doses for hypertension management.^48^ In the present study, no patients had received low doses of CCB. In addition, previous studies have reported that CCB has a stronger effect among younger glaucoma patients with other diseases, such as Flammer syndrome.^49^^–^^52^ By contrast, another recent study showed that CCB use increased the risk for development of POAG.^53^ These conflicting results may again be attributable to the heterogeneity of patients with NTG. Hence, although the effect of CCB may be limited in patients with NTG caused by reasons such as IOP fluctuation or myopia, CCB may be beneficial for patients with NTG attributable to the dysregulation of blood vessels.

This study was subject to several limitations. First, we only investigated structural progression, not functional progression, and chose the inclusion and exclusion criteria to detect structural progression. VF is generally considered to be more informative for detecting progression in moderate to advanced glaucoma,^54^ while OCT is considered more sensitive to the detection of progression in the early stages of the disease.^9^^,^^55^^,^^56^ This difference can be partially ascribed to the difficulty in detecting structural progression in advanced glaucoma due to the floor effect.^55^^,^^57^ Therefore eyes with advanced glaucoma were excluded from this study, and the risk factors and cutoff values identified by our study may not be applicable to advanced glaucoma. Second, only daytime IOP and BP were measured. Although recent studies have reported the influence of nocturnal systemic hypotension on glaucoma progression,^58^^,^^59^ the retrospective nature of this study precluded the obtainment of nocturnal measurements. Nonetheless, our results show that, similar to nocturnal hypotension or nocturnal drops in BP, minimum daytime SBP and DBP could be potential risk factors for glaucoma progression. Third, because of the limited patient records, antihypertensive medication data were only available for 34 eyes. The limited data from which our results were derived may therefore restrict the reproducibility of our study. Further studies with larger sample sizes are warranted to confirm this relationship. Fourth, because of the retrospective design of our study, the sequential relationship between BP/IOP variables and structural progression could not be demonstrated. For example, changes in treatment due to suspected progression may result in IOP fluctuation. In this case, IOP fluctuation does not cause structural progression, but IOP fluctuation may increase due to the change in treatment. To minimize this possibility, we only analyzed IOP and BP values measured before structural progression was detected. Finally, BP was measured in the upper right arm, and the relationship between BP measured in the arm and at the retrobulbar remains unclear. Therefore the perfusion pressure calculated in this study should be considered an approximate value. Future research may benefit from directly measuring the retrobulbar perfusion pressure directly or analyzing the degree of perfusion using instruments such as OCT angiography.

In conclusion, disc hemorrhage, diabetes, and systemic hypotension were associated with structural progression in medically treated NTG eyes. The tree analysis showed that SBP could feature a stronger association with progressive peripapillary RNFL thinning than could DBP and vice versa for progressive macular GCIPL thinning. We also used tree analysis to identify a target BP to prevent structural progression and expect that these results could help to prevent structural progression in NTG eyes.

## Supplementary Material

Supplement 1

Supplement 2

## References

[1] Trivli A, Koliarakis I, Terzidou C, et al. Normal-tension glaucoma: Pathogenesis and genetics. *Exp Ther Med*. 2019; 17: 563–574. 30651837 10.3892/etm.2018.7011 PMC6307418

[2] Kaiser HJ, Flammer J, Graf T, Stumpfig D Systemic blood pressure in glaucoma patients. *Graefes Arch Clin Exp Ophthalmol*. 1993; 231: 677–680. 8299973 10.1007/BF00919280

[3] Chiotoroiu SM, Stefaniu O, Noaghi M, Teodorescu A, Taina L The Role of Systemic Blood Pressure in Glaucoma Progression. *Rom J Ophthalmol*. 2015; 59: 141–147. 26978881 PMC5712958

[4] Flammer J, Orgul S, Costa VP, et al. The impact of ocular blood flow in glaucoma. *Prog Retin Eye Res*. 2002; 21: 359–393. 12150988 10.1016/s1350-9462(02)00008-3

[5] Sohn SW, Song JS, Kee C Influence of the extent of myopia on the progression of normal-tension glaucoma. *Am J Ophthalmol*. 2010; 149: 831–838. 20231010 10.1016/j.ajo.2009.12.033

[6] Drance S, Anderson DR, Schulzer M, Collaborative Normal-Tension Glaucoma Study G Risk factors for progression of visual field abnormalities in normal-tension glaucoma. *Am J Ophthalmol*. 2001; 131: 699–708. 11384564 10.1016/s0002-9394(01)00964-3

[7] Junoy Montolio FG, Wesselink C, Gordijn M, Jansonius NM Factors that influence standard automated perimetry test results in glaucoma: test reliability, technician experience, time of day, and season. *Invest Ophthalmol Vis Sci*. 2012; 53: 7010–7017. 22952121 10.1167/iovs.12-10268

[8] Leung CK, Cheung CY, Weinreb RN, et al. Evaluation of retinal nerve fiber layer progression in glaucoma: a study on optical coherence tomography guided progression analysis. *Invest Ophthalmol Vis Sci*. 2010; 51: 217–222. 19684001 10.1167/iovs.09-3468

[9] Wollstein G, Schuman JS, Price LL, et al. Optical coherence tomography longitudinal evaluation of retinal nerve fiber layer thickness in glaucoma. *Arch Ophthalmol*. 2005; 123: 464–470. 15824218 10.1001/archopht.123.4.464 PMC1941777

[10] Zhang X, Dastiridou A, Francis BA, et al. Baseline Fourier-domain optical coherence tomography structural risk factors for visual field progression in the Advanced Imaging for Glaucoma Study. *Am J Ophthalmol*. 2016; 172: 94–103. 27651070 10.1016/j.ajo.2016.09.015 PMC5121039

[11] Lee KS, Lee JR, Kook MS Optic disc torsion presenting as unilateral glaucomatous-appearing visual field defect in young myopic Korean eyes. *Ophthalmology*. 2014; 121: 1013–1019. 24507857 10.1016/j.ophtha.2013.11.014

[12] Archer J, Robertson DL CTree: comparison of clusters between phylogenetic trees made easy. *Bioinformatics*. 2007; 23: 2952–2953. 17717036 10.1093/bioinformatics/btm410

[13] Hothorn T, Hornik K, Zeileis A Unbiased recursive partitioning: a conditional inference framework. *J Comput Graph Stat*. 2006; 15: 651–674.

[14] Leske MC, Wu SY, Hennis A, Honkanen R, Nemesure B, Group BES Risk factors for incident open-angle glaucoma: the Barbados Eye Studies. *Ophthalmology*. 2008; 115: 85–93. 17629563 10.1016/j.ophtha.2007.03.017

[15] Tham Y-C, Lim S-H, Gupta P, Aung T, Wong TY, Cheng C-Y Inter-relationship between ocular perfusion pressure, blood pressure, intraocular pressure profiles and primary open-angle glaucoma: the Singapore Epidemiology of Eye Diseases Study. *Br J Ophthalmol*. 2018; 102: 1402–1406. 29331952 10.1136/bjophthalmol-2017-311359

[16] Hulin I, Kinova S, Paulis L, Slavkovsky P, Duris I, Mravec B Diastolic blood pressure as a major determinant of tissue perfusion: potential clinical consequences. *Bratisl Lek Listy*. 2010; 111: 54–56. 20429314

[17] Cruickshank JM. Coronary flow reserve and the J curve relation between diastolic blood pressure and myocardial infarction. *BMJ*. 1988; 297: 1227–1230. 3145062 10.1136/bmj.297.6658.1227 PMC1834729

[18] McEvoy JW, Chen Y, Rawlings A, et al. Diastolic blood pressure, subclinical myocardial damage, and cardiac events: implications for blood pressure control. *J Am Coll Cardiol*. 2016; 68: 1713–1722. 27590090 10.1016/j.jacc.2016.07.754 PMC5089057

[19] Senthong V, Kukongviriyapan U, Settasatian N, Settasatian C, Komanasin N Low diastolic blood pressure is associated with a high atherosclerotic burden in patients with obstructive coronary artery disease. *Cardiol J*. 2018; 25: 345–352. 28980283 10.5603/CJ.a2017.0109

[20] Bengtsson B, Leske MC, Hyman L, Heijl A, Early Manifest Glaucoma Trial G Fluctuation of intraocular pressure and glaucoma progression in the early manifest glaucoma trial. *Ophthalmology*. 2007; 114: 205–209. 17097736 10.1016/j.ophtha.2006.07.060

[21] Rao HL, Addepalli UK, Jonnadula GB, Kumbar T, Senthil S, Garudadri CS Relationship between intraocular pressure and rate of visual field progression in treated glaucoma. *J Glaucoma*. 2013; 22: 719–724. 22595936 10.1097/IJG.0b013e318259b0c2

[22] Nouri-Mahdavi K, Hoffman D, Coleman AL, et al. Predictive factors for glaucomatous visual field progression in the Advanced Glaucoma Intervention Study. *Ophthalmology*. 2004; 111: 1627–1635. 15350314 10.1016/j.ophtha.2004.02.017

[23] Lee PP, Walt JW, Rosenblatt LC, Siegartel LR, Stern LS, Glaucoma Care Study G Association between intraocular pressure variation and glaucoma progression: data from a United States chart review. *Am J Ophthalmol*. 2007; 144: 901–907. 17919446 10.1016/j.ajo.2007.07.040

[24] Leske MC, Heijl A, Hyman L, Bengtsson B Early Manifest Glaucoma Trial: design and baseline data. *Ophthalmology*. 1999; 106: 2144–2153. 10571351 10.1016/s0161-6420(99)90497-9

[25] Ishida K, Yamamoto T, Sugiyama K, Kitazawa Y Disk hemorrhage is a significantly negative prognostic factor in normal-tension glaucoma. *Am J Ophthalmol*. 2000; 129: 707–714. 10926977 10.1016/s0002-9394(00)00441-4

[26] Kim M, Kim DM, Park KH, Kim TW, Jeoung JW, Kim SH Intraocular pressure reduction with topical medications and progression of normal-tension glaucoma: a 12-year mean follow-up study. *Acta Ophthalmol*. 2013; 91: e270–275. 23406253 10.1111/aos.12082

[27] Leske MC, Heijl A, Hyman L, et al. Predictors of long-term progression in the early manifest glaucoma trial. *Ophthalmology*. 2007; 114: 1965–1972. 17628686 10.1016/j.ophtha.2007.03.016

[28] Quigley HA, Addicks EM, Green WR, Maumenee AE Optic nerve damage in human glaucoma. II. The site of injury and susceptibility to damage. *Arch Ophthalmol*. 1981; 99: 635–649. 6164357 10.1001/archopht.1981.03930010635009

[29] De Moraes CG, Prata TS, Liebmann CA, Tello C, Ritch R, Liebmann JM Spatially consistent, localized visual field loss before and after disc hemorrhage. *Invest Ophthalmol Vis Sci*. 2009; 50: 4727–4733. 19458330 10.1167/iovs.09-3446

[30] Grieshaber MC, Terhorst T, Flammer J The pathogenesis of optic disc splinter haemorrhages: a new hypothesis. *Acta Ophthalmol Scand*. 2006; 84: 62–68. 16445441 10.1111/j.1600-0420.2005.00590.x

[31] Grieshaber MC, Flammer J. Does the blood-brain barrier play a role in Glaucoma? *Surv Ophthalmol*. 2007; 52(Suppl 2): S115–121. 17998035 10.1016/j.survophthal.2007.08.005

[32] Sharpe GP, Danthurebandara VM, Vianna JR, et al. Optic disc hemorrhages and laminar disinsertions in glaucoma. *Ophthalmology*. 2016; 123: 1949–1956. 27432205 10.1016/j.ophtha.2016.06.001

[33] Kim YD, Han SB, Park KH, et al. Risk factors associated with optic disc haemorrhage in patients with normal tension glaucoma. *Eye (Lond)*. 2010; 24: 567–572. 19648906 10.1038/eye.2009.163

[34] Soares AS, Artes PH, Andreou P, Leblanc RP, Chauhan BC, Nicolela MT Factors associated with optic disc hemorrhages in glaucoma. *Ophthalmology*. 2004; 111: 1653–1657. 15350318 10.1016/j.ophtha.2004.03.023

[35] Grodum K, Heijl A, Bengtsson B Optic disc hemorrhages and generalized vascular disease. *J Glaucoma*. 2002; 11: 226–230. 12140400 10.1097/00061198-200206000-00011

[36] Klein BE, Klein R, Jensen SC Open-angle glaucoma and older-onset diabetes: the Beaver Dam Eye Study. *Ophthalmology*. 1994; 101: 1173–1177. 8035979 10.1016/s0161-6420(94)31191-2

[37] Nakamura M, Kanamori A, Negi A Diabetes mellitus as a risk factor for glaucomatous optic neuropathy. *Ophthalmologica*. 2005; 219: 1–10. 15627820 10.1159/000081775

[38] Chopra V, Varma R, Francis BA, et al. Type 2 diabetes mellitus and the risk of open-angle glaucoma: the Los Angeles Latino Eye Study. *Ophthalmology*. 2008; 115: 227–232.e221. 17716734 10.1016/j.ophtha.2007.04.049 PMC4864602

[39] Newman-Casey PA, Talwar N, Nan B, Musch DC, Stein JD The relationship between components of metabolic syndrome and open-angle glaucoma. *Ophthalmology*. 2011; 118: 1318–1326. 21481477 10.1016/j.ophtha.2010.11.022 PMC3129406

[40] Flammer J, Orgül S, Costa VP, et al. The impact of ocular blood flow in glaucoma. *Progr Retinal Eye Res*. 2002; 21: 359–393. 10.1016/s1350-9462(02)00008-3 12150988

[41] Grieshaber MC, Flammer J. Blood flow in glaucoma. *Current Opinion in Ophthalmology*. 2005; 16: 79–83. 15744136 10.1097/01.icu.0000156134.38495.0b

[42] Chihara E, Liu X, Dong J, et al. Severe myopia as a risk factor for progressive visual field loss in primary open-angle glaucoma. *Ophthalmologica*. 1997; 211: 66–71. 9097306 10.1159/000310760

[43] Sakata R, Aihara M, Murata H, et al. Contributing factors for progression of visual field loss in normal-tension glaucoma patients with medical treatment. *J Glaucoma*. 2013; 22: 250–254. 23059475 10.1097/IJG.0b013e31823298fb

[44] Sung MS, Kang YS, Heo H, Park SW Optic disc rotation as a clue for predicting visual field progression in myopic normal-tension glaucoma. *Ophthalmology*. 2016; 123: 1484–1493. 27157844 10.1016/j.ophtha.2016.03.040

[45] Kitazawa Y, Shirai H, Go FJ The effect of Ca2(+) -antagonist on visual field in low-tension glaucoma. *Graefes Arch Clin Exp Ophthalmol*. 1989; 227: 408–412. 2806924 10.1007/BF02172889

[46] Netland PA, Chaturvedi N, Dreyer EB Calcium channel blockers in the management of low-tension and open-angle glaucoma. *Am J Ophthalmol*. 1993; 115: 608–613. 8488913 10.1016/s0002-9394(14)71458-8

[47] Flammer J, Konieczka K, Flammer AJ The primary vascular dysregulation syndrome: implications for eye diseases. *EPMA J*. 2013; 4: 14. 23742177 10.1186/1878-5085-4-14 PMC3693953

[48] Strenn K, Matulla B, Wolzt M, et al. Reversal of endothelin-1-induced ocular hemodynamic effects by low-dose nifedipine in humans. *Clin Pharmacol Ther*. 1998; 63: 54–63. 9465842 10.1016/S0009-9236(98)90121-7

[49] Gaspar AZ, Flammer J, Hendrickson P Influence of nifedipine on the visual fields of patients with optic-nerve-head diseases. *Eur J Ophthalmol*. 1994; 4: 24–28. 8019119 10.1177/112067219400400105

[50] Konieczka K, Todorova MG, Bojinova RI, Binggeli T, Chackathayil TN, Flammer J Unexpected effect of calcium channel blockers on the optic nerve compartment syndrome. *Klin Monbl Augenheilkd*. 2016; 233: 387–390. 27116489 10.1055/s-0042-102619

[51] Fang L, Turtschi S, Mozaffarieh M The effect of nifedipine on retinal venous pressure of glaucoma patients with the Flammer-Syndrome. *Graefes Arch Clin Exp Ophthalmol*. 2015; 253: 935–939. 25863672 10.1007/s00417-015-3001-7

[52] Flammer J, Konieczka K. The discovery of the Flammer syndrome: a historical and personal perspective. *EPMA J*. 2017; 8: 75–97. 28725290 10.1007/s13167-017-0090-x PMC5486542

[53] Muskens RP, de Voogd S, Wolfs RC, et al. Systemic antihypertensive medication and incident open-angle glaucoma. *Ophthalmology*. 2007; 114: 2221–2226. 17568677 10.1016/j.ophtha.2007.03.047

[54] Sommer A, Katz J, Quigley HA, et al. Clinically detectable nerve fiber atrophy precedes the onset of glaucomatous field loss. *Arch Ophthalmol*. 1991; 109: 77–83. 1987954 10.1001/archopht.1991.01080010079037

[55] Hood DC, Kardon RH. A framework for comparing structural and functional measures of glaucomatous damage. *Prog Retin Eye Res*. 2007; 26: 688–710. 17889587 10.1016/j.preteyeres.2007.08.001 PMC2110881

[56] Kuang TM, Zhang C, Zangwill LM, Weinreb RN, Medeiros FA Estimating Lead Time Gained by Optical Coherence Tomography in Detecting Glaucoma before Development of Visual Field Defects. *Ophthalmology*. 2015; 122: 2002–2009. 26198809 10.1016/j.ophtha.2015.06.015 PMC4581949

[57] Banister K, Boachie C, Bourne R, et al. Can Automated Imaging for Optic Disc and Retinal Nerve Fiber Layer Analysis Aid Glaucoma Detection? *Ophthalmology*. 2016; 123: 930–938. 27016459 10.1016/j.ophtha.2016.01.041 PMC4846823

[58] Charlson ME, de Moraes CG, Link A, et al. Nocturnal systemic hypotension increases the risk of glaucoma progression. *Ophthalmology*. 2014; 121: 2004–2012. 24869467 10.1016/j.ophtha.2014.04.016 PMC4386594

[59] Tokunaga T, Kashiwagi K, Tsumura T, Taguchi K, Tsukahara S Association between nocturnal blood pressure reduction and progression of visual field defect in patients with primary open-angle glaucoma or normal-tension glaucoma. *Jpn J Ophthalmol*. 2004; 48: 380–385. 15295667 10.1007/s10384-003-0071-6

