# Supplementary material for: Risk Factors Associated with Structural Progression in Normal-Tension Glaucoma: Intraocular Pressure, Systemic Blood Pressure, and Myopia
Source: Invest Ophthalmol Vis Sci. 2020 Jul 27;61(8):35. doi: 10.1167/iovs.61.8.35 (PMC7425752; doi:10.1167/iovs.61.8.35)
Supplement: Supplement 1 [file iovs-61-8-35_s001.pdf]

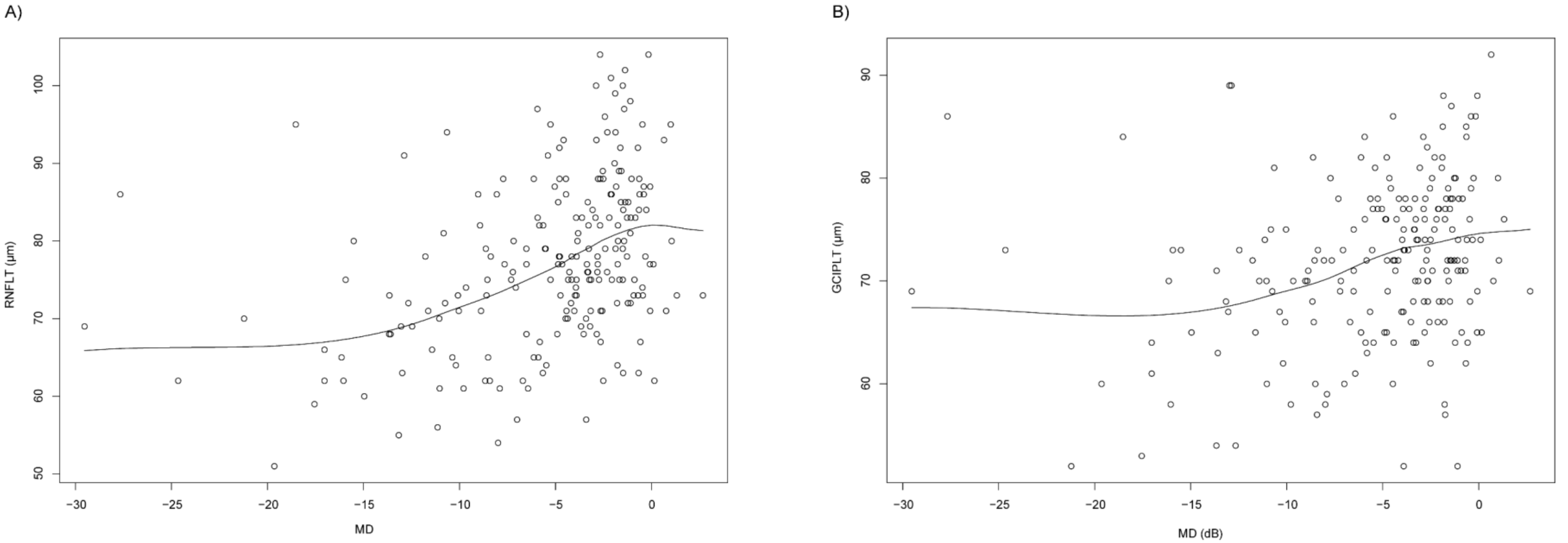

Supplementary Figure 1. A) Scatter plot of mean decibel (MD) and retinal nerve fiber layer thickness (RNFLT). B) Scatter plot of mean decibel (MD) and ganglion cell inner-complex layer thickness (GCIPLT).
