# Supplementary material for: Risk Factors Associated with Structural Progression in Normal-Tension Glaucoma: Intraocular Pressure, Systemic Blood Pressure, and Myopia
Source: Invest Ophthalmol Vis Sci. 2020 Jul 27;61(8):35. doi: 10.1167/iovs.61.8.35 (PMC7425752; doi:10.1167/iovs.61.8.35)
Supplement: Supplement 2 [file iovs-61-8-35_s002.pdf]

Supplementary Table 1. Clinical Variables of Normal and Hypertensive Patients (or Eyes)

	Normal (112 eyes)	HTN (54 eyes)	p^a
**Age, years**	52.2 ±15.5	65.0 ±10.4	<0.001
**Diabetes mellitus, n (%)**	7 (6.2%)	17 (31.5%)	<0.001
**Central corneal thickness (µm)**	535.2 ±38.5	538.0 ±33.8	0.671
**Disc hemorrhage, n (%)**	26 (23.2%)	18 (33.3%)	0.232
**RNFL thickness, µm**	80.9 ± 8.8	80.6 ± 9.2	0.840
**GCIPL thickness, µm**	74.2 ± 5.9	75.1 ± 5.3	0.355
**Mean Deviation, dB**	-4.1 ± 3.6	-4.5 ± 4.2	0.476
**Axial length, mm**	24.9 ± 1.7	23.6 ± 1.3	0.002
**Disc torsion, degrees**	0.9 ± 8.5	-0.1 ± 6.9	0.446
**Disc tilt ratio**	1.2 ± 0.2	1.1 ± 0.2	0.226
**Baseline IOP, mmHg**	15.0 ± 2.4	14.8 ± 2.6	0.657
**Mean IOP, mmHg**	13.2 ± 1.8	12.7 ± 1.9	0.149
**SD IOP, mmHg**	1.5 ± 0.5	1.7 ± 0.5	0.094
**Peak IOP, mmHg**	15.7 ± 2.1	15.3 ± 2.2	0.277
**Mean SBP, mmHg**	121.3 ±12.7	122.6 ±11.8	0.530
**Minimum SBP, mmHg**	109.2 ±13.4	108.5 ±13.6	0.743
**Maximum SBP, mmHg**	134.1 ±14.3	136.5 ±14.0	0.310
**SD SBP, mmHg**	9.8 ± 4.2	10.8 ± 5.1	0.183
**Mean DBP, mmHg**	71.3 ± 8.6	70.6 ± 7.8	0.591
**Maximum DBP, mmHg**	80.7 ± 9.8	80.9 ± 9.5	0.910
**Minimum DBP, mmHg**	62.1 ± 9.9	61.0 ± 8.4	0.481
**SD DBP, mmHg**	7.4 ± 2.9	7.4 ± 3.1	0.864
**MAP, mmHg**	88.0 ± 9.5	87.9 ± 8.8	0.967
**MOPP, mmHg**	49.9 ± 6.3	50.1 ± 5.8	0.810
**SPP, mmHg**	108.2 ±12.8	109.9 ±11.7	0.402
**DPP, mmHg**	58.1 ± 8.5	57.8 ± 7.7	0.820
**Progressive peripapillary RNFL thinning, n (%)**	29 (26.6%)	15 (27.8%)	>0.999
**Progressive macular GCIPL thinning, n (%)**	25 (23.4%)	16 (31.4%)	0.379

Parameters are represented as the mean ± standard deviation or n (%). RNFL, retinal nerve fiber layer; GCIPL, ganglion cell-inner plexiform layer; IOP, intraocular pressure; SD, standard deviation; SBP, systolic Blood pressure; DBP, diastolic blood pressure; MAP, mean arterial pressure; MOPP, mean ocular perfusion pressure; SPP, systolic perfusion pressure; DPP, diastolic perfusion pressure; HTN, hypertension.

a: students' t-test. Values significant at p<.05 are indicated in bold.

Supplementary Table 2 Univariable and Multivariable Cox Analysis of Progressive Peripapillary RNFL or Macular GCIPL Thinning in the Eyes of Patients with HTN

	Univariable Cox Analysis			Multivariable Cox Analysis		
	Hazard Ratio	95% CI	P	Hazard Ratio	95% CI	P
**Age, years**	1.045	0.992-1.100	0.095			
**HTN medication**						
**ARB**	1.722	0.452-6.562	0.426			
**CCB**	0.201	0.052-0.773	**0.020**	0.329	0.072-1.498	0.151
**β - blocker**	1.337	0.326-5.490	0.687			
**Diuretics**	NA	NA	NA			
**# of medication**	0.547	0.226-1.325	0.181			
**Diabetes mellitus, n (%)**	1.699	0.744-3.879	0.208			
**Central corneal thickness (μm)**	1.003	0.986-1.020	0.734			
**Disc hemorrhage, n (%)**	0.991	0.431-2.279	0.984			
**RNFL thickness, μm**	1.010	0.970-1.052	0.639			
**GCIPL thickness, μm**	1.045	0.969-1.126	0.253			
**Mean deviation, dB**	0.985	0.890-1.091	0.773			
**Axial length, mm**	0.996	0.665-1.491	0.985			
**Disc torsion, degrees**	0.924	0.857-0.997	**0.041**	0.990	0.871-1.125	0.873
**Disc tilt ratio**	0.504	0.113-2.244	0.369			
**Baseline IOP, mmHg**	0.952	0.796-1.140	0.594			
**Mean IOP, mmHg**	0.914	0.700-1.193	0.507			
**SD IOP, mmHg**	1.788	0.737-4.341	0.199			
**Peak IOP, mmHg**	0.979	0.811-1.181	0.822			
**Mean SBP, mmHg**	0.953	0.919-0.989	**0.011**	0.930	0.875-0.987	**0.018**
**Minimum SBP, mmHg**	0.947	0.914-0.980	**0.002**			
**Maximum SBP, mmHg**	0.978	0.949-1.008	0.150			
**SD SBP, mmHg**	1.031	0.952-1.116	0.460			
**Mean DBP, mmHg**	0.954	0.901-1.011	0.112			
**Maximum DBP, mmHg**	0.992	0.953-1.033	0.701			
**Minimum DBP, mmHg**	0.923	0.877-0.972	**0.002**			
**SD DBP, mmHg**	1.149	1.021-1.292	**0.021**			
**MAP, mmHg**	0.947	0.900-0.996	**0.034**			
**MOPP, mmHg**	0.923	0.854-0.998	**0.044**			
**SPP, mmHg**	0.954	0.920-0.991	**0.014**			
**DPP, mmHg**	0.957	0.902-1.015	0.141			

RNFL, retinal nerve fiber layer; GCIPL, ganglion cell-inner plexiform layer; HTN, hypertension; ARB, angiotensin II receptor blockers; CCB, calcium channel blocker; IOP, intraocular pressure; SD, standard deviation; SBP, systolic Blood pressure; DBP, diastolic blood pressure; MAP, mean arterial pressure; MOPP, mean ocular perfusion pressure; SPP, systolic perfusion pressure; DPP, diastolic perfusion pressure; CI, confidence interval. Values significant at p<.05 are indicated in bold.

Supplementary Table 3 Univariable and Multivariable Cox Analysis of Progressive Peripapillary RNFL Thinning in Eyes of Patients with HTN

	Univariable Cox Analysis			Multivariable Cox Analysis		
	Hazard Ratio	95% CI	P	Hazard Ratio	95% CI	P
**Age, years**	1.011	0.959-1.065	0.686			
**HTN medication**						
**ARB**	1.684	0.323-8.770	0.536			
**CCB**	0.196	0.039-0.986	**0.048**	0.764	0.096-6.049	0.798
**β - blocker**	0.867	0.166-4.519	0.865			
**Diuretics**	NA	NA	NA			
**# of medication**	0.434	0.118-1.595	0.209			
**Diabetes mellitus, n (%)**	2.393	0.833-6.874	0.105			
**Central corneal thickness (μm)**	1.018	0.993-1.044	0.156			
**Disc hemorrhage, n (%)**	1.938	0.696-5.400	0.205			
**RNFL thickness, μm**	1.030	0.978-1.085	0.270			
**GCIPL thickness, μm**	1.051	0.955-1.156	0.306			
**Mean deviation, dB**	1.107	0.961-1.276	0.160			
**Axial length, mm**	0.848	0.440-1.633	0.621			
**Disc torsion, degrees**	0.926	0.843-1.018	0.110			
**Disc tilt ratio**	0.937	0.212-4.137	0.931			
**Baseline IOP, mmHg**	1.150	0.941-1.404	0.172			
**Mean IOP, mmHg**	0.923	0.645-1.319	0.659			
**SD IOP, mmHg**	2.724	0.825-8.991	0.100			
**Peak IOP, mmHg**	0.993	0.772-1.276	0.954			
**Mean SBP, mmHg**	0.947	0.903-0.993	**0.023**			
**Minimum SBP, mmHg**	0.915	0.871-0.962	**0.001**			
**Maximum SBP, mmHg**	0.981	0.945-1.018	0.299			
**SD SBP, mmHg**	1.076	0.976-1.186	0.142			
**Mean DBP, mmHg**	0.964	0.899-1.034	0.302			
**Maximum DBP, mmHg**	1.006	0.961-1.053	0.787			
**Minimum DBP, mmHg**	0.910	0.850-0.975	**0.008**			
**SD DBP, mmHg**	1.215	1.045-1.412	**0.011**	1.122	0.919-1.371	0.259
**MAP, mmHg**	0.949	0.891-1.010	0.102			
**MOPP, mmHg**	0.926	0.841-1.019	0.115			
**SPP, mmHg**	0.947	0.903-0.994	**0.027**	0.909	0.827-0.999	**0.047**
**DPP, mmHg**	0.966	0.900-1.037	0.336			

RNFL, retinal nerve fiber layer; GCIPL, ganglion cell-inner plexiform layer; HTN, hypertension; ARB, angiotensin II receptor blockers; CCB, calcium channel blocker; IOP, intraocular pressure; SD, standard deviation; SBP, systolic Blood pressure; DBP, diastolic blood pressure; MAP, mean arterial pressure; MOPP, mean ocular perfusion pressure; SPP, systolic perfusion pressure; DPP, diastolic perfusion pressure; CI, confidence interval. Indicated in bold type, p<.05 indicates statistical significance.

Supplementary Table 4 Univariable and Multivariable Cox Analysis of Progressive macular GCIPL Thinning in the Eyes of Patients with HTN

	Univariable Cox Analysis			Multivariable Cox Analysis		
	Hazard Ratio	95% CI	P	Hazard Ratio	95% CI	P
**Age, years**	1.098	1.018-1.184	**0.016**	1.088	1.006-1.176	**0.035**
**HTN medication**						
**ARB**	1.132	0.202-6.338	0.888			
**CCB**	0.646	0.129-3.244	0.595			
**β - blocker**	3.225	0.418-24.890	0.261			
**Diuretics**	NA	NA	NA			
**# of medication**	0.980	0.403-2.381	0.964			
**Diabetes mellitus, n (%)**	0.946	0.323-2.772	0.920			
**Central corneal thickness (μm)**	0.996	0.978-1.014	0.642			
**Disc hemorrhage, n (%)**	1.481	0.523-4.199	0.460			
**RNFL thickness, μm**	1.028	0.975-1.083	0.308			
**GCIPL thickness, μm**	1.065	0.980-1.158	0.137			
**Mean deviation, dB**	0.954	0.851-1.069	0.415			
**Axial length, mm**	1.108	0.715-1.716	0.647			
**Disc torsion, degrees**	0.911	0.820-1.011	0.079			
**Disc tilt ratio**	0.117	0.002-7.089	0.305			
**Baseline IOP, mmHg**	0.956	0.725-1.262	0.753			
**Mean IOP, mmHg**	0.964	0.685-1.357	0.833			
**SD IOP, mmHg**	1.879	0.608-5.805	0.273			
**Peak IOP, mmHg**	0.998	0.769-1.295	0.987			
**Mean SBP, mmHg**	0.976	0.935-1.018	0.258			
**Minimum SBP, mmHg**	0.965	0.927-1.005	0.085			
**Maximum SBP, mmHg**	0.991	0.957-1.024	0.588			
**SD SBP, mmHg**	1.018	0.923-1.123	0.722			
**Mean DBP, mmHg**	0.972	0.906-1.043	0.427			
**Maximum DBP, mmHg**	0.992	0.944-1.943	0.763			
**Minimum DBP, mmHg**	0.925	0.867-0.988	**0.019**	0.935	0.874-1.000	0.050
**SD DBP, mmHg**	1.114	0.962-1.291	0.149			
**MAP, mmHg**	0.970	0.913-1.030	0.323			
**MOPP, mmHg**	0.956	0.873-1.408	0.339			
**SPP, mmHg**	0.976	0.935-1.019	0.267			
**DPP, mmHg**	0.973	0.906-1.045	0.452			

RNFL, retinal nerve fiber layer; GCIPL, ganglion cell-inner plexiform layer; HTN, hypertension; ARB, angiotensin II receptor blockers; CCB, calcium channel blocker; IOP, intraocular pressure; SD, standard deviation; SBP, systolic Blood pressure; DBP, diastolic blood pressure; MAP, mean arterial pressure; MOPP, mean ocular perfusion pressure; SPP, systolic perfusion pressure; DPP, diastolic perfusion pressure; CI, confidence interval. Values significant at $p<.05$ are indicated in bold.
